# Evaluation of the Mineral Status of Two Ecosystems for Sustainable Goat Rearing in the Iberian Peninsula

**DOI:** 10.1155/2012/853548

**Published:** 2012-01-12

**Authors:** David Vilallonga, Antonio Jiménez, Joaquín Sánchez, Santiago Andrés

**Affiliations:** Faculty of Veterinary Sciences, University of Extremadura, Avda. Universidad, 10071 Cáceres, Spain

## Abstract

The mineral status in two ecosystems typical of the Iberian Peninsula was evaluated. Ecosystem I was formed by forests and ecosystem II by hilly areas. The levels of calcium, phosphorus, magnesium, iron, copper, zinc and selenium in soils, rations and serum were measured. The concentratons of iron, copper, zinc and selenium were also checked in liver. Ecosystem I showed higher values of every mineral, except for phosphorus. Seasonal differences were recorded for rations and serum, with higher values in spring. The rations produced by both ecosystems met the mineral requirements of goats in lactation. Thus, both ecosystems are suitable for the development of an ecological goat farming system. However, extra supply of minerals, particularly calcium, may be needed in the maximum productions periods.

## 1. Introduction

Goat farming in the Mediterranean basin has been traditionally very important. This area accounts for an important part of the global goat stock. The importance of goat farming in this part of the world comes from two aspects. The first is the management of marginal areas, which are unsuitable for raising other domestic herbivore species. The second is to help to maintain the rural settlements, which have traditionally lost population due to the lack of local resources [1]. The best way to fulfil these objectives could be to adapt the traditional extensive goat rearing in ecological livestock production.

Ecological livestock rearing is an emerging face of agricultural activity that does not degrade the environment and helps in its sustainability. These benefits have been recognized by the agricultural policy of the European Union (EU), which promotes ecological practices by EU subsidies [2]. In the goat farms of the Mediterranean countries, the main product is milk, which is mostly delivered for cheese making. Suckling and weaned kids are used for meat. These products are successfully marketed, especially if they are ecologically produced. Thus, ecological goat farming could benefit from better prices [3].

In the Southwest of the Iberian Peninsula, the production of domestic herbivores has adapted to the traditional Mediterranean forest, giving rise to two distinct ecosystems. The first one called “*dehesa*” in Spain and “*montado*” in Portugal and the second ecosystem and called “sierra” in Spain and “serra” in Portugal [4].

One of the most limiting factors of these production systems would be to satisfy the requirements for essential minerals, as calcium, phosphorous, magnesium, iron, copper, zinc, and selenium, which play fundamental roles in the metabolism of goats [5]. Other countries ecosystems face similar challenges about potential mineral deficiencies, as arid or semiarid rangelands [6, 7].

The aim of this study was to check, by the evaluation of the mineral status of animals, if these two typically Mediterranean ecosystems could be managed under ecological rules.

## 2. Materials and Methods

### 2.1. Ecosystems

Ecosystem I was formed by forests elevated from 300 and 600 m above sea level that had been modified by woodcutting to favour animal grazing. Ecosystem II included less modified forests, situated in hilly areas elevated from 700 to 1,200 above sea level. The soils in ecosystem I were formed by granite or slate, while in ecosystem II, the soils were predominantly slate.

Both systems presented three main components: trees, bushes, and pasture. The trees were represented by *Quercus ilex* and *Quercus suber*. Bushes were formed by *Retama sphaerocarpa*, *Genista florida*, *Cistus salvifolius,* and *Rosmarinus officinalis*. Pastures included a variety of species, mainly *Lolium spp*, *Bromus spp*, *Trifolium spp,* and *Medicago spp*. In ecosystem II, *Quercus rober* substituted for *Quercus ilex*, the presence of bushes was higher, and pasture was less abundant. The second ecosystem studied presented fewer possibilities for use by livestock than ecosystem I except for goats [1].

The climate in both ecosystems was of the Mediterranean type, characterized by mild winters and dry summers. Rainfall concentrated in spring and autumn. In ecosystem I, the annual mean minimum and maximum temperatures were 10.8°C and 21.4°C, respectively, and the mean rainfall was 523 mm. In ecosystem II, temperatures were approximately two degrees less and the mean rainfall 200 mm more [8].

### 2.2. Farms

Twenty farms were selected for this experiment from the main areas of goat farming in the Southwest of the Iberian Peninsula. Ten of them belonged to ecosystem I, and the same number was representative of ecosystem II. The size of the selected flocks included the typical range in both ecosystems: small (50–200 heads), medium (300–500 heads), and large (600 heads or more). The mean altitude of the farms in ecosystem I was 450 m and in ecosystem II 900 m above sea level. The soils and the vegetation of the farms were characteristic of each ecosystem.

### 2.3. Nutrition and Husbandry

The nutritional practices are influenced by the climatic conditions in the Mediterranean area. There are usually two peaks of herbage growth, a minor peak between October and December after the first autumn rains and a main peak between March and June, when the herbage starts to mature and dry out. However, in ecosystem II, the maturation occurs later, and grazing is possible for longer periods.

The ration consisted of grazing and browsing on leaves and twigs of bushes, with supply of cereals and forages obtained in the same farm to improve milk production. The intake levels are not easily measured, but each goat received 800–1000 g of cereals in lactation and 300–400 g in nonproductive periods. Hay was offered ad libitum during the night. As pasture production in ecosystem I was higher, and bushes were more abundant in ecosystem II, grazing was more intensive in the first ecosystem, and the intake of ligneous vegetation was more elevated in the second ecosystem.

The management practices were very similar in both ecosystems. Goats were reared at range. After the morning milking, when they received concentrates, goats grazed and browsed on leaves. In the evening, they were guided to the shelters for the evening milking, where they were confined during the night. Kids remained in the shelters all the day, and spent the night with the does.

The reproductive management has traditionally followed a scheme of breeding periods at the end of spring and autumn, with kidding in autumn (early kidding) and in spring (late kidding). Nowadays, due to the requirements of the cheese industry, the tendency is to plan reproduction in order to have lactating does at the beginning of the summer. The mean prolificacy was 1.55 kids per doe.

### 2.4. Animals

“Verata” breed does and their offspring were selected for this experiment. The goats of this autochthonous breed are medium size, black, grey or, more commonly, brown. The aptitude of this breed is milk meat and the average milk production reaches 250 litres in 200–300 milking days.

### 2.5. Samples

Soil samples were taken from each distinct part of the farms according to the crop or land profile. These samples were composed of twelve subsamples, performed by randomly probing. Samples were transported in transparent plastic bag and kept at room temperature until analysis.

Sampling of grass was made in the parts of the farms representative of the grazing areas. The number of samples was variable according to the heterogeneity and size of the meadows. Samples of the concentrates were also taken, together with the twigs and leaves of bushes according to the consumption habits of goats. As the diet was influenced by the climatic variations, sampling included the rations of autumn and spring, which were representative of the different types of rations received through the year. Food samples were properly identified and kept at room temperature until further analysis.

Blood samples were taken according to the size of the flock in a proportion of 10%. Blood samples were obtained from does between the second and fifth delivery, selected randomly at weaning time. Samples were withdrawn from the jugular vein, put into clotting tubes, and kept at room temperature until serum separation. Analyses were conducted in the next 24 hours.

Samples from livers were taken at the slaughterhouse from kids weaned 21 days after birth and fed only milk. The number of samples was proportional to the number of animals slaughtered and the size of the flock. Samples were frozen at −40°C until analysis.

### 2.6. Analysis

Minerals were determined in the soils and rations by atomic absorption (Perkin Elmer 550, IZASA, Sevilla, Spain) after the pretreatment of the sample and extraction techniques [9]. Calcium, phosphorus, magnesium, iron, copper, and zinc were assayed in the serum by colorimetric methods (UV160A, Shimazdu, Kyoto, Japan), and selenium was measured by graphite furnace atomic absorption (Perkin Elmer 550). Liver samples were evaluated for iron, copper, zinc and selenium by atomic absorption (Perkin Elmer 550).

### 2.7. Clinical Surveillance in the Farms

An experienced team of observers performed a clinical monitoring of the goats in the experiment in order to detect possible clinical manifestations of mineral deficiency during the last period of gestation and the lactation.

### 2.8. Statistical Analysis

In the calculation of the means values of mineral content in the ration, it has been taken into account the contribution of the different ingredients making up the rations. The grass accounts for 75% of the ration and the concentrates for 25%. Thus, the mean mineral contents of the rations are weighted means and not merely arithmetic means. The differences in the mean concentration of minerals in soils, ration, serum, and liver between the different ecosystems and sampling seasons (spring or autumn) were evaluated by an interactive two-way analysis of variance. The G-stat 2.0 statistical package was used for this study [10].

## 3. Results and Discussion

### 3.1. Variations according to the Ecosystem

The mean concentrations of calcium, magnesium, iron, copper, zinc, and selenium obtained in the soils were significantly higher in the farms of ecosystem I than in the farms of ecosystem II (Table 1). This may be supported by the shallow soils in the hills, which have a lower mineral content than alluvial soils. In addition, the movements of the flocks from the hills to the valleys during centuries may have contributed to the transfer of nutrients from the higher parts to the lower ones [11]. The mean concentration of phosphorus in the soils was significantly higher in ecosystem II (Table 1). This may be related to the erosive phenomena of the rocks in the hills, which are very rich in phosphorus and slowly release their phosphoric content, increasing the concentration of this mineral in the surrounding areas [12].

The differences in the mineral composition of the rations in ecosystem I and ecosystem II resembled the differences in the soils (Table 2). The highest concentrations of all the minerals included in the study, except for phosphorus, were found in ecosystem I. This may be understood from the view of the nutrition practices in ecological livestock production. The rations were based on grazing and browsing on leaves and twigs of bushes, and the supplements were hays and crops coming from the same farms [2]. This was the origin of the close relationship observed between the composition of the soils and the mineral content of the rations.

The highest values in the serum were obtained in ecosystem I, except for phosphorus (Table 3). These results were in accordance with those recorded in the ration. Similar findings were reported by Goff [13], who affirmed that the serum concentration of calcium, phosphorus, and magnesium were related to the composition of the ration. This may be explained by the normal regulatory mechanisms of the mineral concentrations in the biological fluids, where the supply of minerals in the ration plays a main role [14].

The results of iron, copper, zinc, and selenium concentration obtained in the liver samples (Table 4) followed the trend of the ration and serum results. These values were indicative of the mineral supply [15], which was higher in ecosystem I.

### 3.2. Variations according to the Season

The statistical appraisal of the means of mineral concentration in the soils did not reveal significant differences between seasons (Table 1). It is generally considered that the lands not intensively cropped do not present dramatic changes in the soil composition [16]. The agricultural practices performed on the farms of the trial were not intensive at all, given that only a small part of the farms was devoted to forage and grain production for animal feeding.

The mineral content of the ration provided in the farms in autumn was significantly different from the ration supplied in spring, with higher means in the last season (Table 2).

The presence of seasonal variations in the mineral composition of the rations consumed by the goats was indicative of a better supply of minerals in the spring. This finding was in accordance with the changes reported for the mineral concentration of pasture in spring [17]. As the production system of the farms of the study was extensive, most part of the nutrients of the ration was acquired by grazing. Due to the high rainfall and the mild temperatures, in spring there was a good production of good quality grass. On the contrary, in autumn, herbage yield is lower, with less dry matter and a poor mineral concentration [16]. This seasonal variation in the rations is presumably one of the causes of the changes recorded in goat production efficiency [7].

Significant differences between seasons were found for the serum concentrations of all the minerals studied, with higher values in spring (Table 3). Some authors [17, 18] have related such seasonal variations to the effect of the weather on the dry matter and the fibre content of pasture. These factors would contribute to a lesser digestibility of the ration. In addition, the grass in autumn rations had less dry matter and a lower mineral content than the spring grass. Supplements were given in the autumn, but they came from local sources, as prescribed by the rules of ecological livestock production, and did not increase significantly the mineral supply [2].

The concentration of zinc and selenium in the liver was significantly higher in spring, but iron and copper did not present seasonal differences (Table 4). The two last mentioned trace elements have body reserves that permit to maintain the functions related to them during a period of some months [19]. The level of these elements in the liver could be considered as an indicator of the mineral supply in the previous months. Thus, it does not reflect the short-term changes in the mineral content of the ration, produced by the climatic conditions. These determinations should be a complement to serum assays in the assessment of the mineral status in goat farms managed at range [5].

### 3.3. Comparison with the Normal Values in Soil, Ration, Serum, and Liver

The values found for calcium in the soil (Table 1) may be considered low in the both ecosystems studied, especially in ecosystem II [20]. These low concentrations were a consequence of the paedological conditions of the soils in the trial, classified as primary granite sands. These soils are derived from recently formed rocks and have low pH. These factors have been related to a low presence of calcium in the soil [12]. The values of phosphorus, magnesium, and the trace elements were in the normal range in both ecosystems [1, 16]. This finding could have been anticipated, given that these were alluvial soils. The lowest values were found in the hilly areas (ecosystem II), where the deficiency of some trace elements has been previously described [21].

Ecosystem I produced rations that met the requirements of lactation in goats for magnesium and iron, almost covered the need for phosphorus, copper, zinc, and selenium, and did not reach the minimum calcium requirements (Table 2). In ecosystem II, the content of magnesium, iron, and phosphorus in the ration met the lactation requirements, but the opposite was true for calcium, selenium, copper, and zinc [19]. The two last mentioned minerals were also the main mineral imbalances detected in comparable studies [6]. The high content of phosphorus in the ration in ecosystem II is remarkable and is supported by the high content of this element in the soils of the hills. The low content of calcium, together with the normal or high concentration of phosphorus, accounted for a ratio calcium/phosphorus in the ration from 2/1 in ecosystem I, which might be considered tolerable, to 1/4 in ecosystem II, which was clearly unacceptable [13].

Mean serum concentrations of phosphorus, magnesium, iron, and zinc were in the normal range (Table 3). Copper and selenium were around the lower limits, and calcium presented subnormal values [19].

The low concentration of calcium in the soils (Table 1) detected in the two ecosystems produced rations with low content in this mineral (Table 2), which did not comply with the requirements for lactation [19]. Thus, goats consuming these rations showed low levels of calcium in the serum (Table 3).

The adequate values reported for phosphorus and magnesium were the result of the level of these elements in the soils and the ration. In the case of phosphorus, it might relate to the ability of goats to select the vegetal species with the highest concentration in this mineral and the high ratio of phosphorus absorption in this species [22].

The values reported for calcium and phosphorus in the serum yielded a calcium/phosphorus ratio from 1.5/1 to 0.9/1, which may be considered inadequate [13]. As a consequence, some clinical cases of hypocalcemia were reported in the farms of ecosystem II, especially in autumn. The number of cases registered was lower than it could be expected judging from the low calcium values. This low prevalence of hypocalcemia might be explained by the mechanisms of adaptation of the animals to the low mineral supply [23].

The serum concentrations of the trace elements (Table 3) were supported by the mineral composition of the soils (Table 1) and the rations (Table 2). Iron showed normal serum values. This is a common finding in mineral assays performed on goats in other parts of the world, as dietary deficiency of iron has not been reported in goats [24].

Our results of copper, zinc, and selenium in the serum could be considered as the minimum of the normal range [25] but not an indicative of an overall deficiency. The lowest values were found in ecosystem II in autumn, where deficiency values were found. However, no clinical case of copper deficiency was recorded in the farms of our experiment in contrast to the reports from other mountain areas in Spain [21]. However, in the farms of our experiment, the prevalence of foot rot disease was high. The deficiency of zinc causes hyperkeratosis in the stratum corneum of the toe. This disorder, especially under bad weather conditions, makes the foot more susceptible to the invasion by *Fusobacterium necrophorum* [26].

Marginal selenium levels were detected. These values were compatible with the previous history of selenium-related diseases. However, in the interpretation of these results the adaptation to the supply of rations with low selenium content should be taken into account [23], just as the low predisposition of goats to suffer from nutritional myodystrophy [27]. This could explain the absence of clinical cases of this disease.

Iron concentration in the liver was in the normal range. Copper, zinc, and selenium were slightly over the lower limit of the range (Table 4). These values were indicative of the moderate content of these minerals in the soils (Table 1) and, thus, in the rations (Table 2).

## 4. Conclusions

The rations produced by ecosystem I met the mineral requirements of goats in lactation, particularly in spring. Ecosystem II provided rations with lower mineral content, especially in autumn.

Both ecosystems were prepared for the development of ecological goat farming system, but some corrective measures should be applied in the gestation and lactation. These control programmes should be aimed to increase the mineral content of the rations in two ways: fertilizing the soils to enrich the mineral content of the local forages and crops or importing ecologically produced concentrates from areas with adequate mineral concentration in the soils.

## Figures and Tables

**Table 1:** Mean concentration (mean ± SD) of minerals in the soils.

Mineral	Ecosystem				Comparison	
	I		II		Ecosystem	Season
	Spring	Autumn	Spring	Autumn		
Ca (ppm)	656 ± 300	623 ± 200	487 ± 337	439 ± 337	*P* < 0.01	NS
P (ppm)	12.7 ± 1.8	12.5 ± 0.8	15.7 ± 2.2	15.9 ± 1.2	*P* < 0.01	NS
Mg (ppm)	188 ± 32	179 ± 14	143 ± 25	151 ± 17	*P* < 0.01	NS
Fe (ppm)	8.40 ± 1.90	8.30 ± 1.47	5.79 ± 1.50	6.14 ± 1.77	*P* < 0.05	NS
Cu (ppm)	4.25 ± 0.03	4.04 ± 0.09	2.48 ± 0.07	2.59 ± 0.10	*P* < 0.01	NS
Zn (ppm)	10.0 ± 1.35	9.50 ± 1.40	8.55 ± 1.20	8.00 ± 2.00	*P* < 0.05	NS
Se (ppm)	0.26 ± 0.04	0.27 ± 0.05	0.17 ± 0.08	0.16 ± 0.07	*P* < 0.05	NS

**Table 2:** Mean concentration (mean ± SD) of minerals in the rations.

Mineral	Ecosystem				Comparison	
	I		II		Ecosystem	Season
	Spring	Autumn	Spring	Autumn		
Ca (% DM)	0.40 ± 0.08	0.28 ± 0.03	0.16 ± 0.03	0.13 ± 0.02	*P* < 0.001	*P* < 0.05
P (% DM)	0.29 ± 0.08	0.26 ± 0.11	0.44 ± 0.09	0.41 ± 0.15	*P* < 0.01	*P* < 0.05
Mg (% DM)	0.26 ± 0.01	0.20 ± 0.06	0.17 ± 0.08	0.13 ± 0.03	*P* < 0.01	*P* < 0.05
Fe (% DM)	0.47 ± 0.05	0.12 ± 0.07	0.06 ± 0.03	0.03 ± 0.02	*P* < 0.001	*P* < 0.01
Cu (ppm)	11.11 ± 1.16	9.14 ± 2.03	8.85 ± 1.06	8.14 ± 2.27	*P* < 0.01	*P* < 0.05
Zn (ppm)	42.2 ± 1.98	34.2 ± 1.15	30.0 ± 0.69	28.5 ± 0.38	*P* < 0.05	*P* < 0.05
Se (ppm)	0.10 ± 0.03	0.07 ± 0.02	0.06 ± 0.03	0.03 ± 0.01	*P* < 0.01	*P* < 0.05

**Table 3:** Mean concentration (mean ± SD) of minerals in serum.

Mineral	Ecosystem				Comparison	
	I		II		Ecosystem	Season
	Spring	Autumn	Spring	Autumn		
Ca (mg/dL)	8.68 ± 2.18	7.80 ± 1.41	7.45 ± 0.96	5.98 ± 2.34	*P* < 0.01	*P* < 0.01
P (mg/dL)	6.20 ± 2.64	5.28 ± 1.93	7.90 ± 3.87	6.84 ± 1.72	*P* < 0.01	*P* < 0.05
Mg (mg/dL)	2.55 ± 0.54	2.13 ± 0.49	2.06 ± 0.49	1.98 ± 0.39	*P* < 0.05	*P* < 0.05
Fe (*μ*g/dL)	126.0 ± 63.7	120.3 ± 50.2	112.2 ± 52.0	88.77 ± 42.0	*P* < 0.05	*P* < 0.05
Cu (*μ*g/dL)	169.6 ± 44.8	115.9 ± 51.0	150.7 ± 91.9	108.9 ± 52.9	*P* < 0.05	*P* < 0.05
Zn (*μ*g/dL)	75.8 ± 12.5	71.3 ± 12.7	62.1 ± 17.1	50.3 ± 10.1	*P* < 0.01	*P* < 0.05
Se (*μ*g/dL)	16.4 ± 4.6	10.2 ± 2.1	11.3 ± 3.7	8.9 ± 2.9	*P* < 0.05	*P* < 0.05

**Table 4:** Mean concentration (mean ± SD) of trace elements in liver (ppm DM).

Mineral	Ecosystem				Comparison	
	I		II		Ecosystem	Season
	Spring	Autumn	Spring	Autumn		
Fe	402.0 ± 43.7	390.3 ± 30.2	302.2 ± 42.0	288.77 ± 35.0	*P* < 0.05	NS
Cu	90.6 ± 10	85.9 ± 9.8	65.7 ± 9.8	58.9 ± 9.7	*P* < 0.05	NS
Zn	185.8 ± 22.5	171.3 ± 22.3	162.1 ± 23.6	150.3 ± 20.4	*P* < 0.05	*P* < 0.05
Se	1.3 ± 0.4	1.0 ± 0.1	0.9 ± 0.3	0.7 ± 0.2	*P* < 0.05	*P* < 0.05
